# Movement Behaviors and Bone Biomarkers in Young Pediatric Cancer Survivors: A Cross-Sectional Analysis of the iBoneFIT Project

**DOI:** 10.3390/nu16223914

**Published:** 2024-11-16

**Authors:** Jose J. Gil-Cosano, Esther Ubago-Guisado, Francisco J. Llorente-Cantarero, Andres Marmol-Perez, Andrea Rodriguez-Solana, Juan F. Pascual-Gazquez, Maria E. Mateos, Jose R. Molina-Hurtado, Beatriz Garcia-Fontana, Pedro Henrique Narciso, Panagiota Klentrou, Luis Gracia-Marco

**Affiliations:** 1 Department of Health Sciences and Biomedicine, Faculty of Health Sciences, Universidad Loyola Andalucía, 41704 Sevilla, Spain; 2 Department of Physical Education and Sports, Faculty of Sport Sciences, Sport and Health University Research Institute (iMUDS), University of Granada, 18071 Granada, Spain; 3 Instituto de Investigación Biosanitaria, ibs.Granada, 18012 Granada, Spain; 4 Maimonides Biomedical Research Institute of Cordoba (IMIBIC), 14004 Cordoba, Spain; 5 Department of Specific Didactics, Faculty of Education, University of Cordoba, 14071 Cordoba, Spain; 6 CIBEROBN, Biomedical Research Networking Center for Physiopathology of Obesity and Nutrition, Carlos III Health Institute, 28029 Madrid, Spain; 7 Pediatric and Adolescent Hematology and Oncology Service, Pediatrics and Pediatric Surgery Clinical Management Unit, Virgen de las Nieves University Hospital, 18014 Granada, Spain; 8 Pediatric Oncology Unit, Department of Pediatrics, Reina Sofia University Hospital, 14004 Cordoba, Spain; 9 Endocrinology and Nutrition Unit, University Hospital Clínico San Cecilio, 18007 Granada, Spain; 10 CIBER of Frailty and Healthy Aging (CIBERFES), Carlos III Health Institute, 28029 Madrid, Spain; 11 Laboratory of InVestigation in Exercise LIVE, Department of Physical Education, Sao Paulo State University (UNESP), Presidente Prudente 19060-900, Brazil; 12 Department of Kinesiology, Brock University, St. Catharines, ON L2S 3A1, Canada; 13 Centre for Bone and Muscle Health, Brock University, St. Catharines, ON L2S 3A1, Canada

**Keywords:** bone turnover, exercise, children, cancer, myokines, osteokines

## Abstract

Background/Objectives: This study aims to investigate the association of movement behaviors with irisin, sclerostin, and bone turnover markers in young pediatric cancer survivors. Methods: A total of 116 young pediatric cancer survivors (12.1 ± 3.3 years; 42% female) were recruited. Time spent in movement behaviors over at least seven consecutive 24 h periods was measured by accelerometers (wGT3x-BT accelerometer, ActiGraph). Blood samples were collected at rest and serum was analyzed for irisin, sclerostin, cross-linked telopeptide of type I collagen (CTX), procollagen type I amino-terminal propeptide (P1NP), total osteocalcin (OC), alkaline phosphatase (ALP), 25-hydroxyvitamin D, parathyroid hormone (PTH), calcium, phosphorous, and magnesium. Results: Irisin and sclerostin were not significantly correlated with bone turnover markers. Sedentary time was negatively correlated with the P1NP (r = −0.411, *p* = 0.027) and total OC (r = −0.479, *p* = 0.015) Z-scores, whereas moderate-to-vigorous physical activity was positively correlated with the P1NP (r = 0.418, *p* = 0.024) and total OC (r = 0.478, *p* = 0.016) Z-scores. Moreover, total physical activity was positively correlated with the total OC Z-score (r = 0.448, *p* = 0.025). Finally, the uncoupling index [CTX/P1NP] was positively correlated with sedentary time (r = 0.424, *p* = 0.012) and negatively correlated with light physical activity (r = −0.352, 0.041). Conclusions: Reducing sedentary time and increasing physical activity may favor bone formation over resorption in young pediatric cancer survivors.

## 1. Introduction

Cancer survival refers to the time span from diagnosis to the rest of a person’s life [1]. It has experienced a remarkable increase in youth during the last decades in developed countries, with a 5-year survivorship rate of 85% in children and 82% in adolescents [2]. However, cancer treatments are linked to impaired bone mass accretion during childhood and adolescence, increasing the likelihood of osteoporosis later in life [3]. This impairment is caused by an alteration in bone turnover, which during bone accretion must favor bone formation over bone resorption, with bone turnover markers reflecting such alterations in this dynamic process [4].

Physical activity (PA) is known to improve bone mineral density in young survivors of pediatric cancer [5]; however, its role on bone turnover markers has been less investigated. Previous studies have shown that PA seems to induce changes in bone turnover markers in healthy adolescents [6], in part because osteocytes, the transducers of mechanical signal arising from PA, communicate with both osteoclasts and osteoblasts [7]. For instance, higher levels of bone formation markers (procollagen type I N-terminal propeptide [P1NP] and alkaline phosphatase [ALP]) and lower levels of bone resorption markers (collagen type I cross-linked C-telopeptide [CTX] and parathormone [PTH]) have been related to more steps per day in pubertal females [8]. Likewise, moderate-to-vigorous PA (MVPA) has also been associated with higher ALP in pubertal females [9]. In addition, soccer participation is associated with higher levels of P1NP in male pubertal [10] and female adolescent athletes [11], whereas basketball participation is associated with higher levels of total osteocalcin (OC) in female young adults [12], compared to non-osteogenic sport or controls. Similarly, Eliakim et al. [13] found an increase in bone formation (total OC, ALP, and P1NP) and a decrease in bone resorption (N-terminal telopeptide cross-link [NTX]) after a 5-week weight-bearing exercise program in adolescent males. However, following a single plyometric exercise bout, both ALP and NTX increased in prepubertal boys [14]. Regarding young pediatric cancer survivors, Mogil et al. [15] found an increase in the receptor activator of nuclear factor-kappa B ligand, a marker of bone resorption, after a 12-month intervention of whole-body vibration.

Animal studies have also demonstrated that PA stimulates the production of irisin by muscles, which promotes bone remodeling by increasing sclerostin expression in osteocytes [16]. In this regard, Jurimae et al. [17] reported a positive association between irisin and sclerostin in adolescent females. However, another study reported that, unlike their adult counterparts, adolescent swimmers did not show a significant irisin response to a high-intensity interval swimming trial [18]. Likewise, no significant post-exercise changes in sclerostin have been observed in pubertal males and females [14,19]. Sclerostin, a Wnt antagonist secreted by osteocytes, inhibits bone formation by competitively binding to the LRP-5 receptor on bone cells [20]. Additionally, recent studies indicate that sclerostin exerts an indirect catabolic effect on bone by raising the RANK/OPG mRNA ratio, which promotes osteoclastic activity and bone resorption [21]. In this regard, sclerostin has been associated with CTX in adolescent females [17].

Although PA and bone turnover marker levels and their associations have been investigated in healthy children [22], they have been less examined in young pediatric cancer survivors. Specifically, to our best of knowledge, irisin, sclerostin, and their relationships to PA and bone turnover markers are unknown in this population. Therefore, the aim of the study was to investigate the associations of PA with irisin, sclerostin, and bone turnover markers in young survivors of pediatric cancer. It was hypothesized that higher levels of PA would be associated with higher levels of irisin, sclerostin, and bone formation markers and lower levels of bone resorption markers.

## 2. Materials and Methods

### 2.1. Design

This cross-sectional study was conducted under the umbrella of the iBoneFIT project [23]. This trial is a multicenter, parallel-group randomized controlled study designed to assess the impact of a 9-month online exercise program on bone health in young survivors of pediatric cancer. Participants were recruited from Pediatric Oncology and Hematology Units at “Virgen de las Nieves” Hospital in Granada and “Reina Sofia” Hospital in Cordoba, Spain. Eligible participants were aged 6–18 years, free from active cancer treatment, diagnosed at least a year before enrollment, and had prior exposure to radiotherapy and/or chemotherapy. Due to COVID-19, data collection was split into two waves: (a) October 2020 to February 2021, and (b) December 2021 to March 2022.

**Figure 1:**
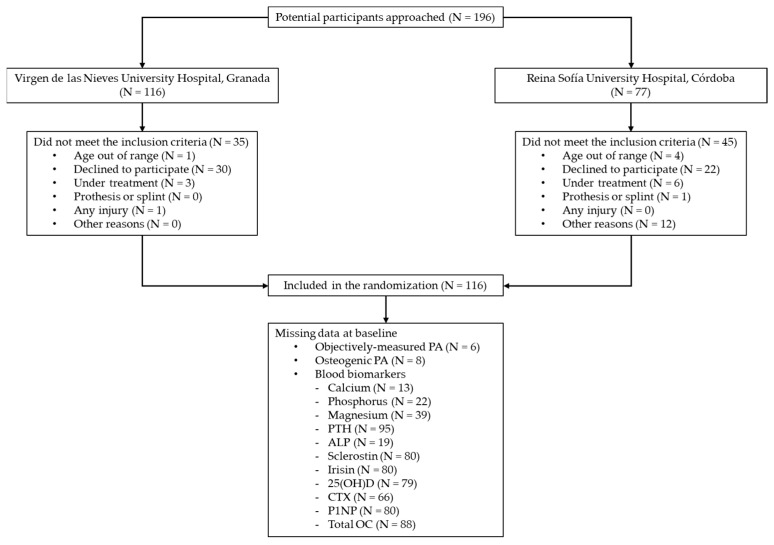
Flowchart of the study. PA, physical activity; PTH, parathyroid hormone; ALP, alkaline phosphatase; CTX, collagen type I cross-linked C-telopeptide; P1NP, procollagen type I N-terminal propeptide; OC, osteocalcin.

Although we recruited 116 young pediatric cancer survivors (12.1 ± 3.3 years old, mean ± SD; 42% female), the sample sizes slightly vary for some variables due to missing data (i.e., some survivors were unable to perform some of the tests, or declined a particular test during their assessment) (Figure 1).

Written informed consent from parents and assent from survivors were obtained prior to trial participation. The iBoneFIT project received approval from the Ethics Committee on Human Research of the Regional Government of Andalusia (Reference: 4500, 19 December 2019). In addition, an amendment of the trial protocol was approved to obtain one more blood tube for bone biomarkers (Reference: 4500, 1 December 2021). The iBoneFIT project adhered to the ethical standards outlined in the Declaration of Helsinki (2013 revision). The trial was registered at isrctn.com (Reference: isrctn61195625, 2 April 2020) and is reported following the STROBE checklist (see Supplementary Table S1) [24].

### 2.2. Measures

#### 2.2.1. Anthropometrics and Somatic Maturity

Participants’ weights were measured using an electronic scale (SECA 861, Hamburg, Germany) with an accuracy of 100 g. Height (cm) was assessed with a precision stadiometer, while sitting height was measured with a SECA 225 (Hamburg, Germany) to the nearest 0.1 cm. Body mass index (BMI) was calculated by dividing body mass (kg) by height (m^2^), and participants were categorized as overweight or obese based on sex- and age-specific BMI cut-offs established by Cole et al. [25]. Somatic maturity was measured using the prediction of years before (negative values) or after (positive values) peak height velocity using validated algorithms for boys and girls [26].

#### 2.2.2. Clinical Data

Medical record abstraction yielded information regarding diagnoses, the duration from treatment completion to baseline data collection, and treatment exposures (radiotherapy, chemotherapy, and/or surgery, whether used individually or in combination). Due to collinearity with treatment exposure, diagnosis was excluded from the analysis. The time since treatment completion was treated as a continuous variable, while treatment exposure was classified as a dichotomous variable (radiotherapy: yes/no). Daily calcium intake (mg) was assessed using a validated food frequency questionnaire [27].

#### 2.2.3. Movement Behaviors

Total PA, MVPA, and light PA (LPA), and sedentary behavior (SB) were measured using the wrist-worn triaxial ActiGraph wGT3x-BT accelerometer (ActiGraph GT3X, Pensacola, FL, USA) for seven consecutive days (24 h/d). Participants were instructed to wear the devices continuously on their nondominant wrist, except during water activities. The accelerometers were set to a sampling frequency of 90 Hz, and the raw data were processed using the GGIR R open-source package. The Euclidean norm of the raw acceleration minus one G was calculated, with negative values rounded to zero, along with the angle of the device’s z-axis, to estimate physical activity parameters [28]. Non-wear time was determined using the standard deviation (SD) of the raw accelerations recorded across the three axes of the accelerometer, as described in previous studies [29]. This non-wear time was then imputed based on the accelerations recorded on the remaining days within the same time window. Appropriate thresholds were used to identify PA intensities and SB (i.e., MVPA: 200 mili-g [mg], LPA: 35–200 mg, SB: 35 mg) [30]. A day was considered valid if the accelerometer recorded data for at least 23 h, and participants were required to wear it for a minimum of 16 h. Only participants with at least one valid day (only one survivor) were included, and sensitivity analyses indicated similar outcomes when compared to survivors with at least three valid weekdays and one weekend day. Daily averages for MVPA, LPA, and SB were calculated as the mean over all 7 days. The wGT3x-BT accelerometer has been previously validated in a young population [31].

Using the bone-specific physical activity questionnaire, the osteogenic activity was reported by the participants considering which sport they had practiced throughout their lifespan and the last year. This tool has been validated to assess the osteogenic characteristics of previous sports and physical activities on the skeleton [32].

#### 2.2.4. Blood Analyses

Samples of venous blood were taken in the morning after fasting overnight. Serum samples were stored at −80 °C until they were analyzed. Calcium (mg/dL), phosphorous (mg/dL), magnesium (mg/dL), PTH (pg/mL), ALP (U/L), total OC (μg/L), and 25-hydroxyvitamin D [25(OH)D, ng/mL] were analyzed following the same laboratory protocol at the Clinical Analysis Unit of each University Hospital. Specifically, 25(OH)D was determined with the two-site immunoassay (Roche Diagnostics SL, Barcelona, Spain). The intra- and inter-assay precision coefficients of variation were 6.9% and 7.2% for 25(OH)D.

CTX (μg/L), P1NP (μg/L), irisin, and sclerostin were analyzed at the Instituto de Investigación Biosanitaria of Granada following standard protocols. CTX was analyzed by enzyme immunoassay (Elecsys b-CrossLaps; Roche Diagnostics, Basel, Switzerland), and P1NP was analyzed by immunoassay on an autoanalyzer COBAS 601 (Roche Diagnostics, Basel, Switzerland). The intra- and inter-assay precision coefficients of variation were 2.0% and 2.9% for CTX, and 5.1% and 6.5% for P1NP. Irisin was determined using an ELISA kit using a specific irisin/FDNC5 monoclonal antibody (R&D Systems Inc., Minneapolis, MN, USA). This assay had intra- and inter-assay CVs of 2.5 and 8.7%, respectively, and the lowest detection limit was 0.25 ng/mL. Sclerostin was analyzed by using a quantitative sandwich ELISA developed by Biomedica (Vienna, Austria). Two samples of known concentrations were tested six times to assess intra-assay variability (4%), and two samples of known concentrations were tested in three assays to assess inter-assay variability (3%). Sclerostin measurements are reported throughout in picograms per milliliter, and lower limit of detection was less than 36.7 pg/mL.

Using reference data of healthy children and adolescents [33], the age- and sex-specific Z-scores of CTX, P1NP, and total OC were calculated. In order to assess the resorption and formation processes of bone remodeling, two uncoupling indexes were calculated: the ratio of CTX to P1NP, and the ratio of CTX to total OC [34]. A positive uncoupling index indicates that bone remodeling was unbalanced in favor of resorption. A negative uncoupling index indicates an imbalance favoring formation.

#### 2.2.5. Statistical Analysis

The descriptive characteristics of the participants are presented as the mean ± standard deviation (SD) or percentages. All variables were checked for normality using the Shapiro–Wilk test and a visual check of the histograms, Q-Q, and box plots.

We utilized the method established by Bieglmayer and Kudlacek [35] to calculate the balance and rate of bone turnover as indirect indicators of the overall effect on bone. This approach compares the multiples of medians (MoMs) for formation markers (P1NP or total OC) and resorption markers (CTX), providing a measure of how much an individual’s results deviate from the median. Specifically, the MoM for each marker was computed using serum concentrations. The bone turnover balance was calculated using the following formula: Bone Turnover Balance = MoM_F_/MoM_R_, where MoM_F_ represents the MoM of P1NP (or total OC) as the formation marker, and MoM_R_ denotes the MoM of CTX as the resorption marker [35]. Additionally, the MoM values were also used to calculate the bone turnover rate, i.e., how fast or slow the turnover occurs, based on the following equation: Bone Turnover Rate = √(MoM_F_^2^ + MoM_R_^2^) [35]. This standardization method represents the balance of bone formation and resorption, along with the rate of bone turnover, though it does not directly represent the bone remodeling unit and is not a direct assessment at the tissue level.

Bivariate correlation analysis was performed to examine the relationships of sclerostin and irisin levels with the CTX, P1NP, and total OC Z-scores. Likewise, bivariate correlation analysis was conducted to examine the relationships of PA variables with the uncoupling indexes.

All the analyses were performed using the IBM SPSS Statistics for Windows version 20.0 (IBM Corp: Armonk, NY, USA), and the level of significance was set to *p* < 0.05.

## 3. Results

The distribution of cancer types for the whole sample is shown in Supplementary Table S2. Most participants were diagnosed with acute lymphoblastic leukemia (38.8%), lymphoma (12.1%), and central nervous system tumors (9.5%).

Descriptive characteristics of our sample are presented by sex in Table 1. Briefly, the mean age was 12.1 ± 3.3 years, and 42.2% were female. Among of them, 17.9% met the PA recommendations, 7.9% met the calcium intake recommendations, and 35.1% presented 25(OH)D sufficiency. Regarding bone turnover markers, the averages were as follows: CTX Z-score = −0.08 ± 0.09; P1NP Z-score = −0.15 ± 0.15; and total OC Z-score = −0.18 ± 0.14.

**Figure 2:**
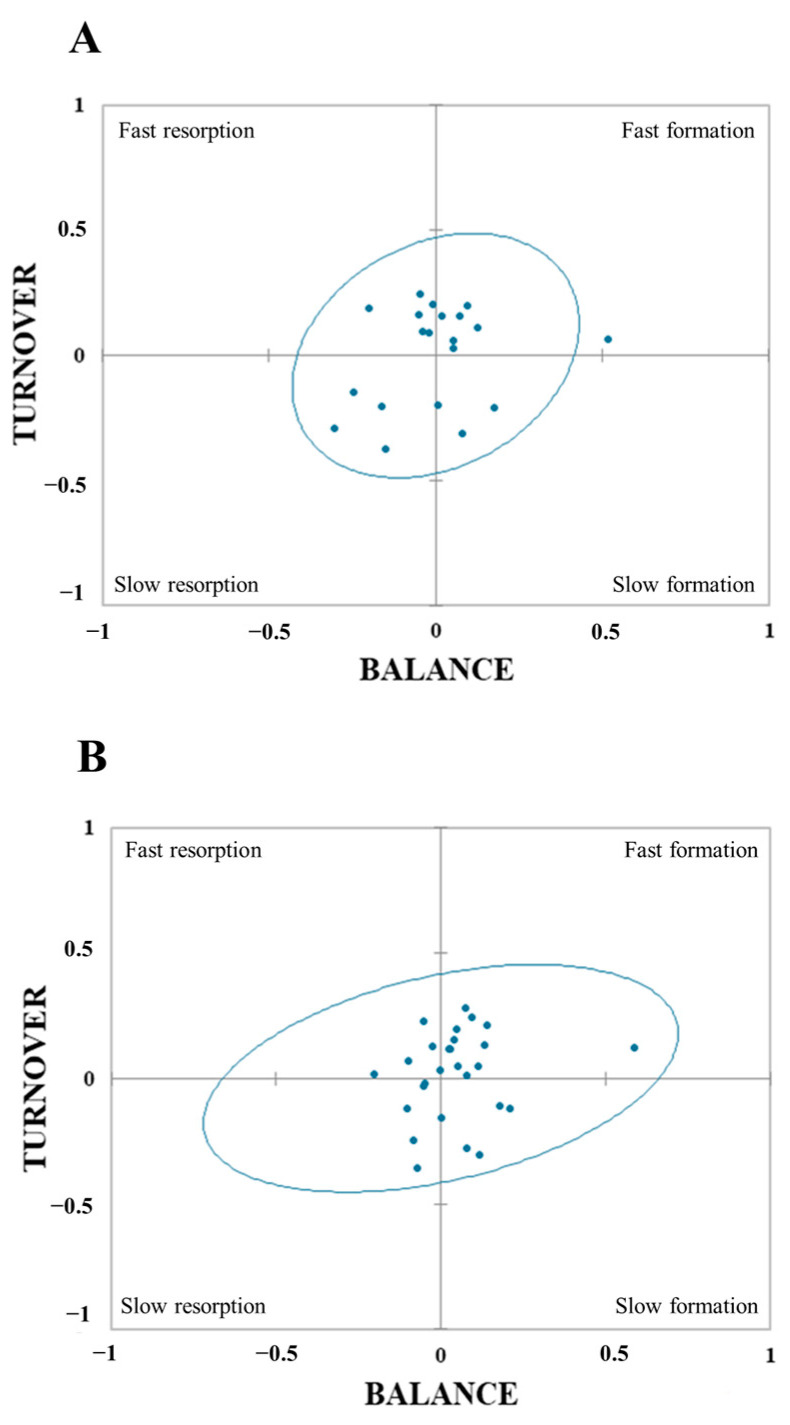
Bone marker plot with 95% confidence ellipsis from (**A**) CTX/P1NP and (**B**) CTX/OC.

The four-field plots are depicted in Figure 2. There were differences in confidence ellipses based on either the CTX/P1NP or CTX/total OC data. The distributions of participants among the features of fast resorption, fast formation, slow formation, and slow resorption for CTX-P1NP (and for CTX-total OC in brackets) were 30% (19.2%), 35% (42%), 15% (15.4%), and 20% (23.1%), respectively.

Concentrations of resorption and formation markers were mathematically transformed to obtain the balance and rate of bone turnover. Four-field plots were built up and the reference data of bone turnover were represented by 95% confidence ellipses [35]. The upper left field of the graph represents dominant resorption together with high turnover. The left bottom field symbolizes slow resorption. The right bottom field corresponds to slow formation. The upper right field typifies fast bone formation.

Table 2 shows correlations between the movement behavior variables, irisin, sclerostin, and bone biomarkers. SB was negatively correlated with the P1NP (Table 2; r = −0.411, *p* = 0.027) and total OC (Table 2; r = −0.479, *p* = 0.015) Z-scores. On the contrary, MVPA was positively correlated with the P1NP (Table 2; r = 0.418, *p* = 0.024) and total OC (Table 2; r = 0.478, *p* = 0.016) Z-scores. Moreover, total PA was positively correlated with the total OC Z-score (Table 2; r = 0.448, *p* = 0.025). Finally, no correlation was found between any of the movement behavior variables and sclerostin or irisin.

**Figure 3:**
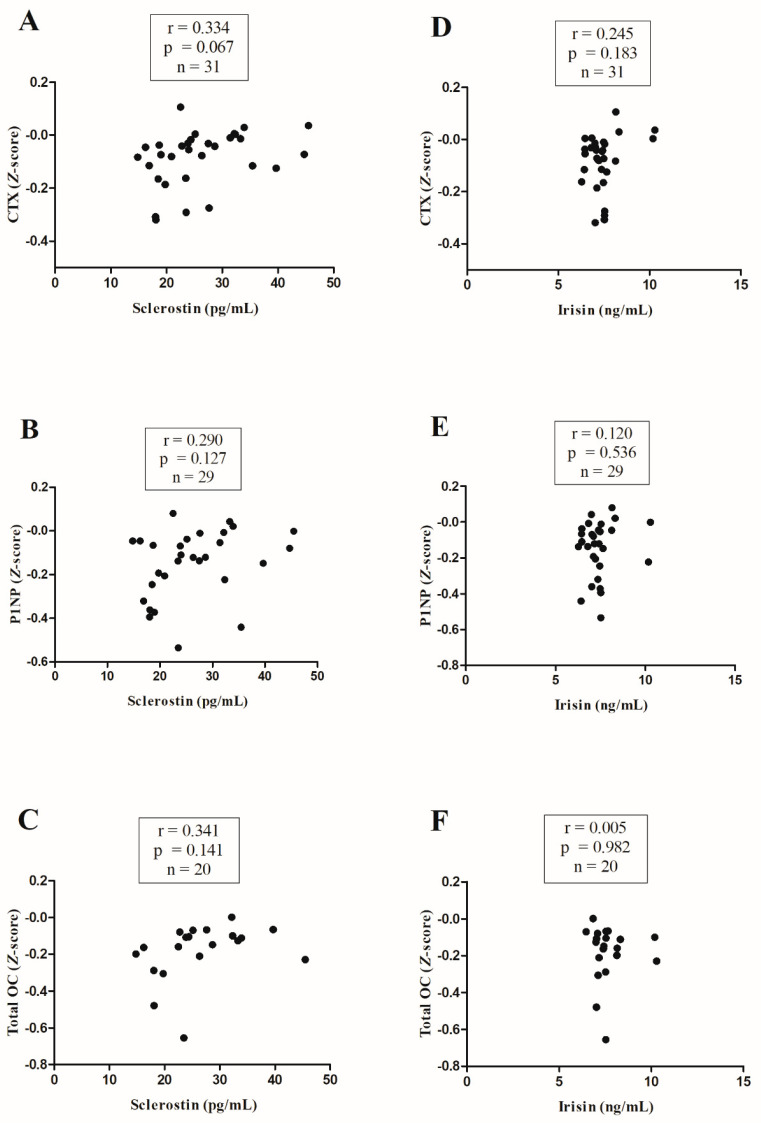
Relationships of circulating sclerostin (panel (**A**–**C**)) and irisin (panel (**D**–**F**)) with bone turnover markers. CTX, collagen type I cross-linked C-telopeptide; P1NP, procollagen type I N-terminal propeptide; OC, osteocalcin.

**Figure 4:**
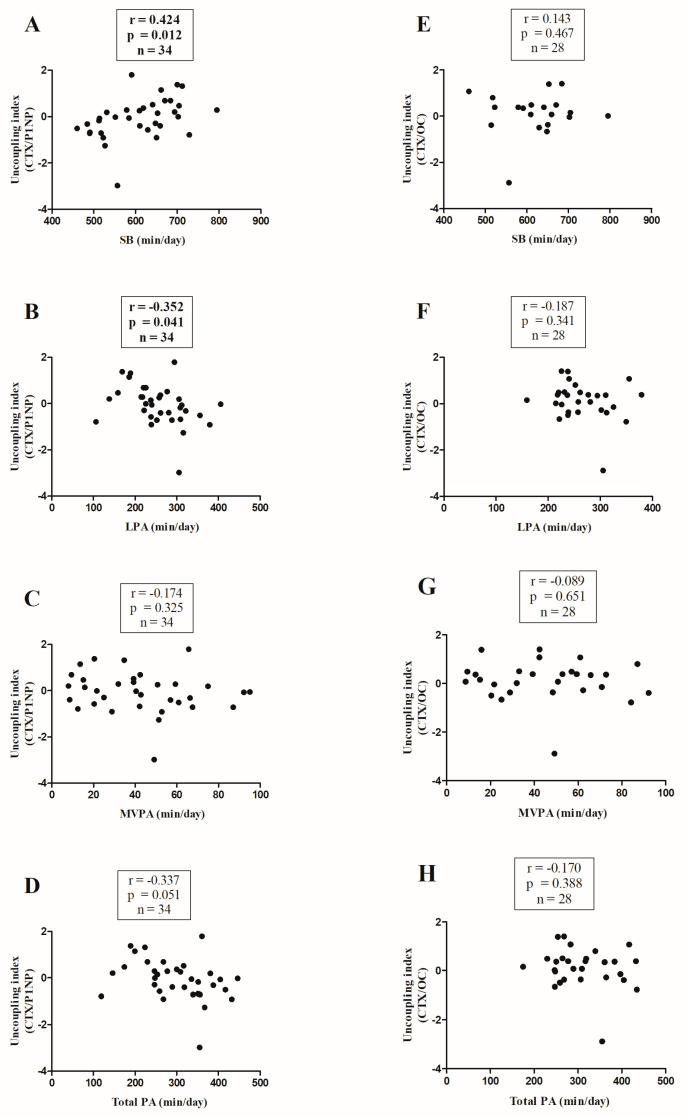
Relationships between movement behaviors and the uncoupling index CTX/P1NP (panel (**A**–**D**)) and the uncoupling index CTX/OC (panel (**E**–**H**)). SB, sedentary behavior; LPA, light physical activity; MVPA, moderate-to-vigorous physical activity; CTX, collagen type I cross-linked C-telopeptide; P1NP, procollagen type I N-terminal propeptide; OC, osteocalcin. Boldface indicates statistical significance.

Bivariate correlation analyses of sclerostin and irisin with movement behaviors are shown in Figure 3. Sclerostin and irisin were not significantly correlated with the CTX, P1NP, or total OC Z-scores (Figure 3). Finally, SB was positively correlated with the uncoupling index [CTX/P1NP] (Figure 4A; r = 0.424, *p* = 0.012), whereas LPA was negatively correlated with the uncoupling index (Figure 4B; r = −0.352, *p =* 0.041).

## 4. Discussion

The main finding of this study was that higher levels of sedentary time were associated with lower levels of bone formation markers (i.e., P1NP, total OC, and ALP), whereas higher levels of LPA, MVPA, and total PA were associated with higher levels of bone formation in young pediatric cancer survivors. Of note, higher levels of LPA and total PA were associated with PTH levels, a marker that calcium is released from the bone into circulation and therefore contributes to bone resorption. Moreover, the associations between the PA variables and the uncoupling index show that sedentary time favored bone resorption, whereas LPA favored bone formation throughout the bone remodeling cycle. Finally, irisin and sclerostin were not associated with either bone turnover markers or PA variables.

As expected, since childhood and adolescence are stages of rapid growth and bone turnover [37], the present data are characterized by fast bone formation and resorption, with bone formation being dominant over resorption in the CTX/P1NP ratio (30 vs. 35%) and the CTX/total OC ratio (19.2 vs. 42%). The lower difference between CTX and P1NP compared to CTX and total OC, might be due to the total OC endocrine actions on the human body (i.e., glucose homeostasis, musculoskeletal functioning, brain development, male fertility, hepatic steatosis, and arterial calcification) [38]. The total OC encompasses carboxylated and undercarboxylated forms, with the undercarboxylated form having several endocrine functions which promote a higher expression from osteoblasts. These results together suggest that bone modeling in young pediatric cancer survivors was occurring as usual in healthy children and adolescents; however, the resting levels of each bone turnover marker were below the sex- and age-normative values.

Our results showed no association of irisin and sclerostin with bone turnover markers. This finding partially disagreed with a previous study that found a positive association between sclerostin and CTX levels in adolescent girls [17]. This could be explained by the lower levels of sclerostin in our sample of young pediatric cancer survivors (mean 101.4 vs. 117.9 pg/mL), which might influence the catabolic activity on the bone. Going through this pathway, irisin has been positively related to sclerostin in female adolescents [17]. In this sense, our sample presented lower levels of irisin compared to previous studies in children and adolescents (mean 7.4 vs. 13.2 and 16.2 ng/mL, respectively) [39], which might compromise sclerostin expression in osteocytes [16].

Despite irisin being a myokine secreted by muscles in response to PA in animal studies, we did not find any association between PA variables and irisin levels in young survivors of pediatric cancer. In agreement with our results, Cai et al. [40] observed no association between PA variables and irisin levels in healthy children. This finding could be attributed to the overall low PA levels of our participants, who only accumulated 41.7 ± 25.8 min/day of MVPA and of whom only 18% met the PA recommendations for children and adolescents. In this regard, a previous study has shown that only strenuous exercise promotes irisin expression in children [41].

A novel and interesting finding of the present study was that sedentary time was negatively associated with bone formation markers (i.e., P1NP, total OC, and ALP), whereas PA levels were positively associated with the same bone formation markers. Additionally, sedentary time and LPA levels were positively and negatively associated with the uncoupling index (CTX-P1NP), respectively. Altogether, increasing mechanical loading through PA, even at light intensity, favored bone formation in young survivors of pediatric cancer. These results are in line with Kambas et al. [8] who reported higher levels of P1NP and ALP in healthy girls who practiced more PA compared to those who practiced less PA (pedometer, steps/day). On the contrary, Pimentel et al. [42] found no association of PA (2-axis accelerometer, MET/day) with P1NP and total OC levels in children with normal weight. We speculate that survivors of pediatric cancer could improve their bone metabolism by routine PA more easily due to higher muscle mass and testosterone in response to PA, as well as PA levels at baseline. In this sense, mechanical loading of skeletal muscle stimulates osteocytes, which in turn induce osteoblast activity [43].

In our study, MVPA levels showed the strongest association with bone formation markers. Generally, vigorous intensities exert higher benefits on bone health than moderate or moderate-to-vigorous intensities in children and adolescents [44]. Likewise, cross-sectional and longitudinal evidence in athletic populations has reported that sport participation involving high mechanical loading (e.g., gymnasts, decathletes, and soccer players) showed higher levels of bone formation and resorption markers than participation in lower impact sports (e.g., swimmers) [11,45]. However, osteogenic PA variables (past and current) were not associated with any bone marker in our sample. The missing effect of osteogenic PA on bone metabolism could be attributed to the fact that young pediatric cancer survivors participate less in organized sports, receive less social support to engage in PA, and perceive themselves as less competitive in physical education at school after treatment [46,47].

Higher LPA and MVPA levels were related to higher PTH levels in young survivors of pediatric cancer. This finding agrees with the results of Pimentel et al. [42], who observed higher PTH in children and adolescents who performed more PA (two-axis accelerometer, MET/day). Intermittent increases in PTH have been shown to result in enhanced bone formation in animal studies [48]. Nevertheless, this result should be taken with caution, since only 35.1% of our sample had sufficient 25(OH)D levels. Of note, circulating calcium and phosphorus levels were under the homeostatic limits considering participants’ age [49,50].

### Strengths and Limitations

The present study has several limitations that should be noted. First, the cross-sectional design prevents the establishment of cause-and-effect relationships, meaning that the findings reported here must be confirmed in prospective studies. Second, the sample size of participants with complete data for all variables studied is relatively small. Third, the participants included in the study were those who chose to enroll in an exercise intervention aimed at improving a bone mineral density. As a result, they may not represent all young pediatric cancer survivors, which could lead to selection bias in the reported compliance rates for physical activity recommendations. The present study also has strengths, such as the use of relevant markers of bone turnover, calcium metabolism, myokines, and osteokines. Furthermore, a triaxial accelerometer was used to objectively measure PA.

## 5. Conclusions

In this cross-sectional study with young pediatric cancer survivors, higher levels of sedentary time and PA (LPA, MVPA, and total PA) are associated with lower and higher levels of bone formation markers (P1NP, total OC, and ALP), respectively. Furthermore, LPA and total PA are positively related with PTH levels. In light of these results, replacing sedentary time with a more active lifestyle, even for LPA, seems to be of great importance to optimize bone modeling after pediatric cancer treatment completion. Future intervention studies should be conducted to confirm these findings in this population.

## Figures and Tables

**Table 1:** Descriptive characteristics of the participants included in the study.

	Total	N	Girls	N	Boys	N
Age (years)	12.1 (3.3)	116	12.2 (3.5)	49	12.0 (3.2)	67
Body mass (kg)	46.6 (18.0)	116	45.2 (18.3)	49	47.6 (17.9)	67
Stature (cm)	147.5 (17.1)	116	145.3 (16.0)	49	149.0 (17.7)	67
Body mass index (*Z*-score)	0.9 (1.1)	116	0.8 (1.1)	49	1.0 (1.2)	67
Underweight	3.5	4	6.1	3	1.5	1
Normal weight	61.2	71	65.4	32	58.2	39
Overweight	20.7	24	16.3	8	23.9	16
Obese	14.6	17	12.2	6	16.4	11
Years from peak height velocity	−0.8 (2.7)	116	0.0 (2.9)	49	−1.3 (2.5)	67
Time from treatment completion (years)	5.0 (3.8)	113	5.2 (4.1)	48	4.9 (3.6)	65
Radiotherapy exposure (yes/no, %)	28/72, 38.9	116	24/76, 31.6	49	30/70, 42.9	67
**Movement behaviors and nutrition**						
SB (min/day)	624.9 (103.0)	110	621.1 (107.2)	48	627.8 (100.5)	62
LPA (min/day)	256.3 (64.0)	110	261.3 (74.4)	48	252.4 (54.9)	62
MVPA (min/day)	41.7 (25.8)	110	36.4 (25.2)	48	45.8 (25.7)	62
Total PA (min/day)	297.9 (84.0)	110	9.7 (13.6)	48	298.2 (75.8)	62
Meeting PA recommendations (yes/no, %)	25/85, 17.9	110	9/39, 18.4	48	16/46, 23.9	62
Osteogenic PA since birth	6.8 [0.3–18.5]	108	5.3 [0.2–13.5]	45	9.0 [0.7–20.6]	63
Calcium intake (mg/day)	785.5 (437.2)	116	702.2 (388.6)	49	846.4 (462.9)	67
Meeting calcium recommendations (yes/no, %)	11/105, 7.9	116	3/46, 6.1	49	8/59, 11.9	67
**Bone metabolism**						
Calcium (mg/dL)	9.8 (0.5)	103	9.8 (0.5)	42	9.9 (0.5)	61
Phosphorus (mg/dL)	4.4 (0.7)	94	4.3 (0.7)	39	4.5 (0.7)	55
Magnesium (mg/dL)	1.9 (0.2)	77	1.9 (0.2)	34	1.9 (0.2)	43
PTH (pg/mL)	46.4 (17.8)	21	50.9 (20.4)	8	43.6 (16.2)	13
ALP (U/L)	224.77 (102.6)	97	191.6 (96.4)	42	250.1 (100.7)	55
Sclerostin (pg/mL)	101.4 (58.0)	36	108.7 (82.6)	16	95.8 (26.8)	20
Irisin (ng/mL)	7.4 (0.9)	36	7.5 (0.9)	16	7.3 (0.8)	20
25(OH)D (ng/mL)	19.9 (8.7)	37	19.2 (8.2)	17	20.5 (9.2)	20
Sufficiency (n, %)	13, 35.1	37	6, 35.3	17	7, 35	20
**Bone turnover markers**						
CTX (μg/L)	1.5 (0.5)	50	1.3 (0.4)	23	1.7 (0.5)	27
P1NP (μg/L)	490.9 (218.9)	36	415.1 (210.2)	16	551.5 (211.3)	20
Total OC (μg/L)	71.1 (32.4)	28	56.5 (34.2)	11	80.5 (28.2)	17
CTX (*Z*-score)	−0.08 (0.09)	45	−0.10 (0.11)	21	−0.07 (0.09)	24
P1NP (*Z*-score)	−0.15 (0.15)	31	−0.17 (0.17)	14	−0.14 (0.14)	17
Total OC (*Z*-score)	−0.18 (0.14)	25	−0.24 (0.19)	10	−0.15 (0.08)	15

Data are presented as the mean (standard deviation), median (interquartile range), or as frequencies (associated percentages), as indicated. SB, sedentary behavior; LPA, light physical activity; MVPA, moderate-to-vigorous physical activity; PA, physical activity; PTH, parathyroid hormone; ALP, alkaline phosphatase; CTX, collagen type I cross-linked C-telopeptide; P1NP, procollagen type I N-terminal propeptide; OC, osteocalcin. World Health Organization physical activity recommendations: ≥60 min/day MVPA. International Osteoporosis Foundation calcium intake recommendations: ≥1000 mg/day for participants between 6 and 8 years old; ≥1300 mg/day for participants older than 8 years. Vitamin D status was defined as follows [36]: Sufficiency, ≥20 ng/mL; Insufficiency, <12 ng/mL.

**Table 2:** Bivariate correlations between physical activity, calcium intake, and bone biomarkers.

	CTX (Z)	P1NP (Z)	Total OC (Z)	ALP	PTH	25(OH)D	Irisin	Sclerostin
SB	0.005	−0.411 *	−0.479 *	−0.350 *	−0.418	0.177	0.193	−0.130
LPA	−0.117	0.257	0.367	0.279 *	0.521 *	−0.323	0.002	0.090
MVPA	0.156	0.418 *	0.456 *	0.374 **	0.405	−0.038	−0.219	0.051
Total PA	−0.043	0.336	0.433 *	0.330 *	0.541 *	−0.251	−0.067	−0.088
Osteogenic PA (since birth)	−0.101	−0.097	0.275	−0.022	−0.091	−0.001	−0.125	−0.087
Osteogenic PA (last year)	−0.072	0.026	0.007	−0.030	−0.214	−0.020	−0.003	−0.067
Sclerostin	0.334	0.290	0.341	0.123	0.115	0.082	0.234	-

SB, sedentary behavior; LPA, light physical activity; MVPA, moderate-to-vigorous physical activity; PA, physical activity; PTH, parathyroid hormone; ALP, alkaline phosphatase; CTX, collagen type I cross-linked C-telopeptide; P1NP, procollagen type I N-terminal propeptide; OC, osteocalcin. Boldface indicates statistical significance. * *p* < 0.050. ** *p* < 0.001.

## Data Availability

The original contributions presented in the study are included in the article/supplementary material, further inquiries can be directed to the corresponding author due to EU-G. The main outcome of the study has not been reported/published yet.

## Supplementary Materials

The following supporting information can be downloaded at https://www.mdpi.com/article/10.3390/nu16223914/s1, Table S1. STROBE Statement—Checklist of items that should be included in reports of *cross-sectional studies*. Table S2. Distribution of cancer types of participants included in this study.
