# Supplementary material for: Movement Behaviors and Bone Biomarkers in Young Pediatric Cancer Survivors: A Cross-Sectional Analysis of the iBoneFIT Project
Source: Nutrients. 2024 Nov 16;16(22):3914. doi: 10.3390/nu16223914 (PMC11597045; doi:10.3390/nu16223914)
Supplement: Supplementary file 1 [file nutrients-16-03914-s001.zip › nutrients-3269479-supplementary.pdf]

Movement behaviors and bone biomarkers in young pediatric cancer survivors: a cross-sectional analysis of the iBoneFIT project

Supplementary Materials

Supplementary Table S1. STROBE Statement—Checklist of items that should be included in reports of *cross-sectional studies*

	Item No	Recommendation	Page No
**Title and abstract**	1	(*a*) Indicate the study's design with a commonly used term in the title or the abstract	1
		(*b*) Provide in the abstract an informative and balanced summary of what was done and what was found	1
**Introduction**			
**Background/rationale**	2	Explain the scientific background and rationale for the investigation being reported	1-2
**Objectives**	3	State specific objectives, including any prespecified hypotheses	2
**Methods**			
**Study design**	4	Present key elements of study design early in the paper	2
**Setting**	5	Describe the setting, locations, and relevant dates, including periods of recruitment, exposure, follow-up, and data collection	2
**Participants**	6	(*a*) Give the eligibility criteria, and the sources and methods of selection of participants	2
**Variables**	7	Clearly define all outcomes, exposures, predictors, potential confounders, and effect modifiers. Give diagnostic criteria, if applicable	2-3
**Data sources/ measurement**	8	For each variable of interest, give sources of data and details of methods of assessment (measurement). Describe comparability of assessment methods if there is more than one group	2-3
**Bias**	9	Describe any efforts to address potential sources of bias	Not applicable
**Study size**	10	Explain how the study size was arrived at	2-3
**Quantitative variables**	11	Explain how quantitative variables were handled in the analyses. If applicable, describe which groupings were chosen and why	3-4
**Statistical methods**	12	(*a*) Describe all statistical methods, including those used to control for confounding	4
		(*b*) Describe any methods used to examine subgroups and interactions	Not applicable
		(*c*) Explain how missing data were addressed	Not applicable
		(*d*) If applicable, describe analytical methods taking account of sampling strategy	
		(*e*) Describe any sensitivity analyses	Not applicable
**Results**			
**Participants**	13	(a) Report numbers of individuals at each stage of study—eg numbers potentially eligible, examined for eligibility, confirmed eligible	Not applicable

		included in the study, completing follow-up, and analysed	
		(b) Give reasons for non-participation at each stage	Not applicable
		(c) Consider use of a flow diagram	Not applicable
**Descriptive data**	14	(a) Give characteristics of study participants (eg demographic, clinical, social) and information on exposures and potential confounders	Table 1, Supplementary Table 2
		(b) Indicate number of participants with missing data for each variable of interest	Table 1, Supplementary Table 2 and Figure 2
**Outcome data**	15	Report numbers of outcome events or summary measures	Table 1
**Main results**	16	(*a*) Give unadjusted estimates and, if applicable, confounder-adjusted estimates and their precision (eg, 95% confidence interval). Make clear which confounders were adjusted for and why they were included	Figure 1, 2, 3 and Supplementary Table 3
		(*b*) Report category boundaries when continuous variables were categorized	Not applicable
		(*c*) If relevant, consider translating estimates of relative risk into absolute risk for a meaningful time period	Not applicable
**Other analyses**	17	Report other analyses done—eg analyses of subgroups and interactions, and sensitivity analyses	Supplementary Table 3
**Discussion**			
**Key results**	18	Summarise key results with reference to study objectives	7-8
**Limitations**	19	Discuss limitations of the study, taking into account sources of potential bias or imprecision. Discuss both direction and magnitude of any potential bias	9
**Interpretation**	20	Give a cautious overall interpretation of results considering objectives, limitations, multiplicity of analyses, results from similar studies, and other relevant evidence	7-9
**Generalisability**	21	Discuss the generalisability (external validity) of the study results	7-9
**Other information**			
**Funding**	22	Give the source of funding and the role of the funders for the present study and, if applicable, for the original study on which the present article is based	13

Note: An Explanation and Elaboration article discusses each checklist item and gives methodological background and published examples of transparent reporting. The STROBE checklist is best used in conjunction with this article (freely available on the Web sites of PLoS Medicine at http://www.plosmedicine.org/, Annals of Internal Medicine at http://www.annals.org/, and Epidemiology at http://www.epidem.com/). Information on the STROBE Initiative is available at www.strobe-statement.org.

Supplementary Table S2. Distribution of cancer types of participants included in this study.

	Total		Girls		Boys	
	**%**	**N**	**%**	**N**	**%**	**N**
Acute lymphoblastic leukaemia	38.8	45	36.7	18	40.3	27
Lymphoma	12.1	14	12.2	6	11.9	8
Central nervous system	9.5	11	10.2	5	9.0	6
Renal tumour	7.8	9	4.1	2	10.5	7
Neuroblastoma	6.9	8	12.2	6	3.0	2
Malignant bone tumour	6.9	8	8.2	4	6.0	4
Histiocytosis	5.2	6	6.1	3	4.5	3
Soft tissue and other extraosseous sarcomas	4.3	5	0.0	0	7.5	5
Retinoblastoma	3.5	4	4.1	2	3.0	2
Hepatic tumour	2.6	3	4.1	2	1.5	1
Other malignant epithelial neoplasms	1.7	2	2.0	1	1.5	1
Unspecified malignant neoplasms	0.9	1	0.0	0	1.5	1
